# Solanesol Ameliorates Anxiety-like Behaviors via the Downregulation of Cingulate T Cell-Restricted Intracellular Antigen-1 in a Complete Freund’s Adjuvant-Induced Mouse Model

**DOI:** 10.3390/ijms251810165

**Published:** 2024-09-21

**Authors:** Shufan Ding, Yifan Li, Zhichao Chen, Jingnan Hu, Jiayi Li, Junlan Li, Yongjie Wang

**Affiliations:** 1 School of Pharmacy, Hangzhou Normal University, Hangzhou 311121, China; 2021211505130@stu.hznu.edu.cn (S.D.); 2021112012225@stu.hznu.edu.cn (Z.C.); 2021211505010@stu.hznu.edu.cn (J.H.); 2021211505031@stu.hznu.edu.cn (J.L.); 2021211505132@stu.hznu.edu.cn (J.L.); 2 School of Basic Medical Sciences, Hangzhou Normal University, Hangzhou 311121, China; 2020211505036@stu.hznu.edu.cn

**Keywords:** solanesol, anxiety disorder, TIA1, neuro-inflammation, anterior cingulate cortex

## Abstract

Anxiety disorder is a universal disease related to neuro-inflammation. Solanesol has shown positive effects because of its anti-inflammatory, anti-tumor, and anti-ulcer properties. This study focused on determining whether solanesol could ameliorate anxiety-like behaviors in a mouse model of neuro-inflammation and identify its working targets. Complete Freund’s adjuvant (CFA)-induced mice that were intra-peritoneally administered with solanesol (50 mg/kg) for 1 week showed a statistically significant reduction in anxiety-like behaviors, as measured by open field and elevated plus-maze tests. Western blot analysis revealed that CFA-induced upregulation of the levels of pro-inflammatory cytokines interleukin (IL)-1β and tumor necrosis factor α (TNF-α), which played crucial roles in regulating anxiety, returned to normal in the anterior cingulate cortex (ACC) after solanesol treatment. The level of T cell-restricted intracellular antigen-1 (TIA1), a key component of stress granules, also decreased in the ACC. Moreover, immunofluorescence results indicated that solanesol suppressed CFA-induced microglial and astrocytic activation in the ACC. CFA was injected in the hind paws of TIA1^Nestin^ conditional knockout (cKO) mice to confirm whether TIA1 is a potential modulatory molecule that influences pro-inflammatory cytokines and anxiety-like behaviors. Anxiety-like behaviors could not be observed in cKO mice after CFA injection with IL-1β and TNF-α levels not remarkedly increasing. Our findings suggest that solanesol inhibits neuro-inflammation by decreasing the TIA1 level to reduce IL-1β and TNF-α expression, meanwhile inhibiting microglial and astrocytic activation in the ACC and ultimately ameliorating anxiety-like behaviors in mice.

## 1. Introduction

Anxiety is a psychiatric disorder characterized by negative physical symptoms and can even trigger the onset of other diseases, such as sleep disorder [1], cardiovascular disease [2], extreme worry, and attention difficulties [3]. If left untreated, anxiety disorder tends to chronically recur [4]. However, traditional anxiolytic medications such as benzodiazepines have many side effects, including dizziness, decreased alertness and concentration, physical dependence, and withdrawal [5,6]. Furthermore, most drugs focus on inhibiting neuronal activation without addressing microglial activity induced by pro-inflammatory cytokine expression. This oversight results in poor treatment outcomes, as the chronic activation of microglia continuously and detrimentally influences neurons [7,8]. Therefore, identifying novel anxiolytic drugs with fewer side effects is necessary.

Solanesol, a long-chain poly-isoprenoid alcohol compound with nine isoprene units, is widely considered an intermediate to ubiquinone drugs. Numerous studies have demonstrated that solanesol plays an important role in resistance to bacteria, fungi, viruses, cancer, inflammation, and ulcers. In addition to these effects, solanesol and its derivatives possess anti-oxidant and anti-tumor properties and show effects on vascular disease, bone rarefaction, and acquired immunodeficiency syndrome [9]. Notably, solanesol protects against neuro-inflammation by decreasing IL-1β and TNF-α levels, providing a significant and positive role in neuroprotection [10,11]. However, previous studies have largely ignored its anxiolytic effects and potential pathways for decreasing pro-inflammatory cytokines in neuroinflammation.

Evidence supports that inflammation plays an essential role in the pathogenesis of mood [12]. Recent studies indicated that pro-inflammatory cytokines, especially TNF-α and IL-1β, are neural biomarkers in anxiety disorders [13,14]. Furthermore, TIA1, an RNA-binding protein, is reportedly associated with inflammation, which is manifested by regulating IL-1β and TNF-α levels [15]. Moreover, pro-inflammatory cytokines (TNF-α and IL-1β), which are linked to microglia and can activate microglia [16,17,18], and the ACC are associated with anxiety [19,20]. However, whether the ratios of TIA1 and pro-inflammatory cytokines are directly or inversely proportional to each other in the ACC during the anti-anxiety process is unclear.

Here, we aimed to determine whether solanesol can reduce IL-1β and TNF-α levels by regulating TIA1 in the ACC, thereby improving anxiety-like behaviors in mice.

## 2. Results

### 2.1. Solanesol Ameliorated Anxiety-like Behaviors in Mice

**Figure 1:**
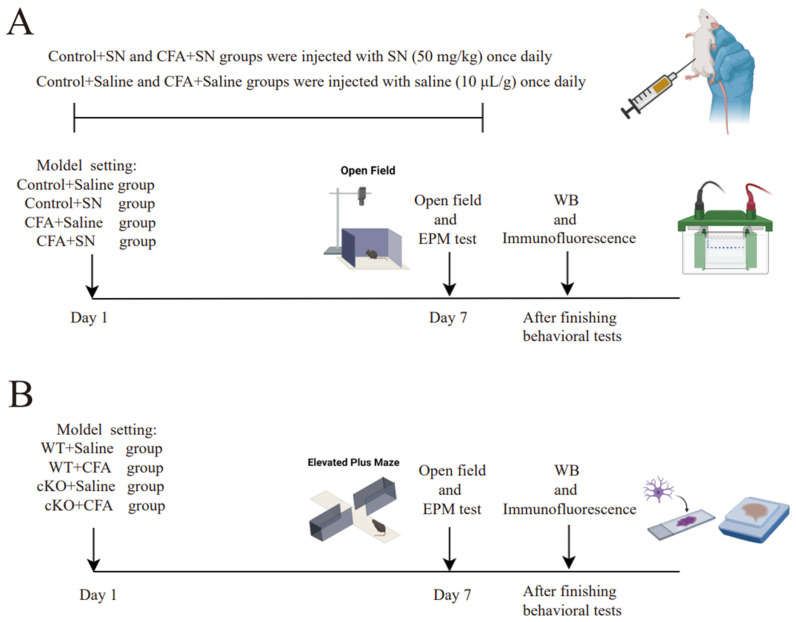
Detailed plan and mouse model parameters of the two experiments; (**A**) We executed the first experiment according to the detailed mouse model parameters, which are as follows. The two control groups were not injected with CFA (40 μL) but saline (40 μL) in the plantar surface of the hind paw. The control+saline group received daily intra-peritoneal injections of saline (10 μL/g); the control+solanesol group was injected daily with solanesol (50 mg/kg) intra-peritoneally; the CFA+saline group received an intra-plantar CFA (40 μL) injection and daily intra-peritoneal saline injections (10 μL/g); and the CFA+solanesol group received an intra-plantar CFA (40 μL) injection and daily intra-peritoneal solanesol (50 mg/kg) injections. The behavior tests began 1 h after the final injection of solanesol was administered. All mice used in the experiment (**A**) were C57BL/6 mice. (**B**) We used cKO mice in the second experiment. The detailed mouse model parameters for the experiment (**B**) are as follows: mice in the WT+saline and WT+CFA groups were wild-type mice and mice in the cKO+CFA and cKO+saline groups were TIA1^Nestin^ cKO mice. The WT+saline and cKO+saline groups both received an intra-plantar saline (40 μL) injection, whereas the other two groups received a CFA (40 μL) injection. This figure was created with BioRender.com (partly adapted from ‘Mouse Behavioral Tests: Anxiety & Depression’, by BioRender.com [2024]. Retrieved from https://app.biorender.com/biorender-templates (accessed on 9 September 2024). Abbreviations: CFA, complete Freund’s adjuvant; SN, solanesol; WB, Western blot; EPM, elevated plus-maze; cKO, TIA1^Nestin^ knockout; TIA1, T cell-restricted intracellular antigen-1; TIA1^Nestin^, TIA1 knockout in Nestin-positive cells; WT, wild-type.

**Figure 2:**
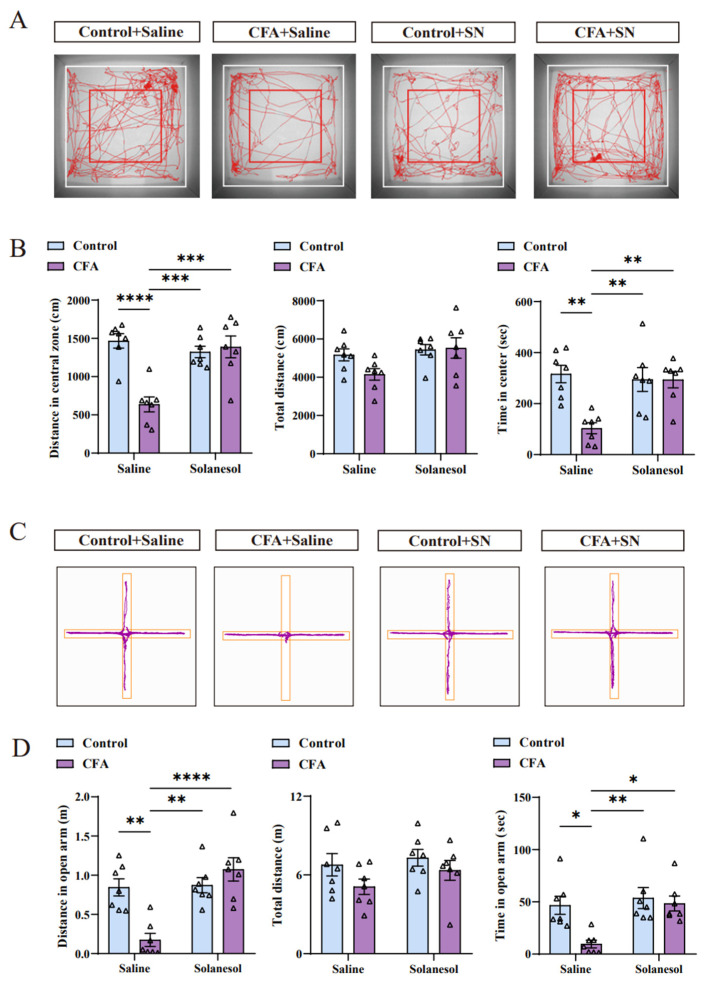
Solanesol ameliorated the anxiety-like behaviors induced by CFA injection. (**A**,**B**) Solanesol treatment significantly increased the number of foot tracks in the red square box (central zone) and inside the white square box (total zone), as indicated in the tracking map (**A**). Based on the detailed data (**B**), the distance traveled in the central zone was significantly decreased after CFA injection, which was ameliorated by solanesol, whereas the total distances did not differ significantly. Moreover, times in the central zone were similar in the context of distance. Solanesol injection had no influence on the behaviors of normal control mice. Statistical analysis was performed using two-way ANOVA (followed by Bonferroni’s multiple comparisons test). Values are expressed as mean ± S.E.M. (n = 7/group; ** *p* < 0.01, *** *p* < 0.001, **** *p* < 0.0001). (**C**,**D**) Cross-track plot of the elevated plus-maze test (**C**) shows that the movements of mice in the open arm in the solanesol group were similar to those in the control group. Based on the specific data (**D**), the time and distance in the open arm both increased after solanesol treatment, which was significantly decreased after CFA injection. As in the open-field test, solanesol did not change the behaviors of control mice in the elevated plus-maze test. Statistical analysis was performed using two-way ANOVA (followed by Bonferroni’s multiple comparisons test). Values are expressed as mean ± S.E.M. (n = 7/group; * *p* < 0.05, ** *p* < 0.01, **** *p* < 0.0001). Abbreviations: ANOVA, analysis of variance; CFA, complete Freund’s adjuvant; and SN, solanesol.

The behavioral tests were performed 1 h after the final solanesol and saline injections on day 7 (Figure 1A). The distances and durations spent in the central area in the open field test were significantly less in CFA group mice compared with mice in the other three groups. Similarly, CFA-injected mice spent less time in the open arm in the elevated plus-maze test compared with mice in other groups. In general, signs of anxiety disorder were indicated by decreased distance and time spent in the central area of the open field test and decreased time spent in the open arm of the elevated plus maze test. Unlike the CFA group, the CFA+solanesol group did not exhibit anxiety-like behaviors in both tests (Figure 2A–D). This finding indicates that solanesol improves anxiety-like behaviors induced by CFA injections in mice. Furthermore, solanesol did not affect the behaviors of the mice that did not receive CFA injections (control+solanesol group).

### 2.2. Solanesol Inhibited the Neuro-Inflammation Response in the ACC 

**Figure 3:**
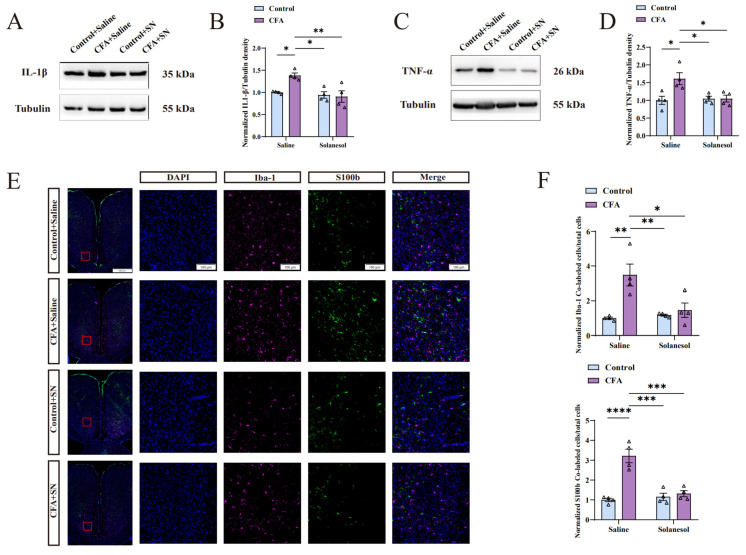
Solanesol mitigated the neuro-inflammation induced by CFA in the ACC. (**A**) Representative Western blot analysis results for IL-1β. (**B**) Normalized density ratio of IL-1β to β-tubulin, which shows that solanesol reduced the CFA-induced increase in IL-1β levels. Statistical analysis was performed using two-way ANOVA (followed by Bonferroni’s multiple comparisons test). Solanesol did not affect IL-1β levels of control mice without CFA injection. Values are expressed as mean ± S.E.M. (n = 4/group; * *p* < 0.05, ** *p* < 0.01). (**C**) Representative Western blot analysis results for TNF-α. (**D**) Normalized density ratio of TNF-α to β-tubulin, which shows that solanesol decreases TNF-α levels after CFA injection. Statistical analysis was performed using two-way ANOVA (followed by Bonferroni’s multiple comparisons test). Solanesol also had no influence on the TNF-α levels of control mice. Values are expressed as mean ± S.E.M. (n = 4/group; * *p* < 0.05). (**E**) Sections were immune-stained for the microglial marker Iba-1 (red) and the astrocytic marker S100b (green), and nuclei were stained with DAPI (blue). Scale bar for the 16 smaller figures on the right = 100 μm; scale bar for the remaining four larger figures on the left = 500 μm. Red boxes in the larger figures represent the locations of the 16 smaller figures in the ACC. (**F**) The ratio of cells with Iba-1 and DAPI co-localization and cells with S100b and DAPI co-localization to the total number of cells indicates the degree of microglial and astrocytic activation. Solanesol clearly reduced microglial and astrocytic activation in the ACC of CFA-injected mice, although it had no influence on normal microglial and astrocytic activation in control mice, indicating that solanesol ameliorates anxiety-like behaviors by inhibiting microglial and astrocytic activation. Statistical analysis was performed using two-way ANOVA (followed by Bonferroni’s multiple comparisons test). Values are expressed as mean ± S.E.M. (n = 4/group; * *p* < 0.05, ** *p* < 0.01, *** *p* < 0.001, and **** *p* < 0.0001). Abbreviations: ACC, anterior cingulate cortex; ANOVA, analysis of variance; CFA, complete Freund’s adjuvant; IL, interleukin; TNF-α, tumor necrosis factor α; SN, solanesol; DAPI, 4′,6-diamidino-2-phenylindole; Iba-1, ionized calcium-binding adapter molecule 1; S100b, S100 calcium-binding protein B; kDa, kilodalton.

After the behavior tests, we conducted the Western bolt experiments as previously described [21,22]. Emerging research has shown that pro-inflammatory cytokines are affected in anxiety disorders [23]. Therefore, we hypothesized that the anti-anxiety activity of solanesol would be associated with decreased pro-inflammatory cytokine levels in the ACC. After extracting ACC protein samples for Western blot analysis, we found that CFA injection increased IL-1β and TNF-α levels. However, IL-1β and TNF-α expression were reduced in the CFA+solanesol group, with no difference observed in the WT+saline and WT+solanesol groups (Figure 3A–D), suggesting that solanesol ameliorates anxiety-like behaviors by downregulating IL-1β and TNF-α in the ACC and had no influence on IL-1β and TNF-α expression in mice without neuro-inflammation.

### 2.3. Solanesol Treatment Inhibited Microglial and Astrocytic Activation in the ACC of CFA-Injected Mice

Microglia and astrocytes have unique roles in regulating inflammatory responses in the central nervous system [24]. The biomarkers for astrocytes and microglia are S100b and Iba-1, respectively. We determined the number of S100b- and Iba-1-positive cells in the control, CFA, control+solanesol, and CFA+solanesol groups. Moreover, after counting the number of cell nuclei stained via DAPI, microglia stained via Iba-1, and astrocytes stained via S100b, we calculated the number of the two types of co-labeled cells (those stained by both DAPI and Iba-1 or DAPI and S100b). The ratio of co-labeled cells and cell nuclei indicates the degree of microglial and astrocytic activation, which was significantly augmented after CFA injection, although the degree of microglial and astrocytic activation in the CFA+solanesol group did not differ from that in the normal control level (Figure 3E,F). Furthermore, solanesol did not disturb the natural activation of microglia and astrocytes. These results proved that solanesol treatment inhibits microglial and astrocytic activation in the ACC.

### 2.4. Solanesol Mitigated the Neuro-Inflammation Response in the ACC by Downregulating TIA1 

**Figure 4:**
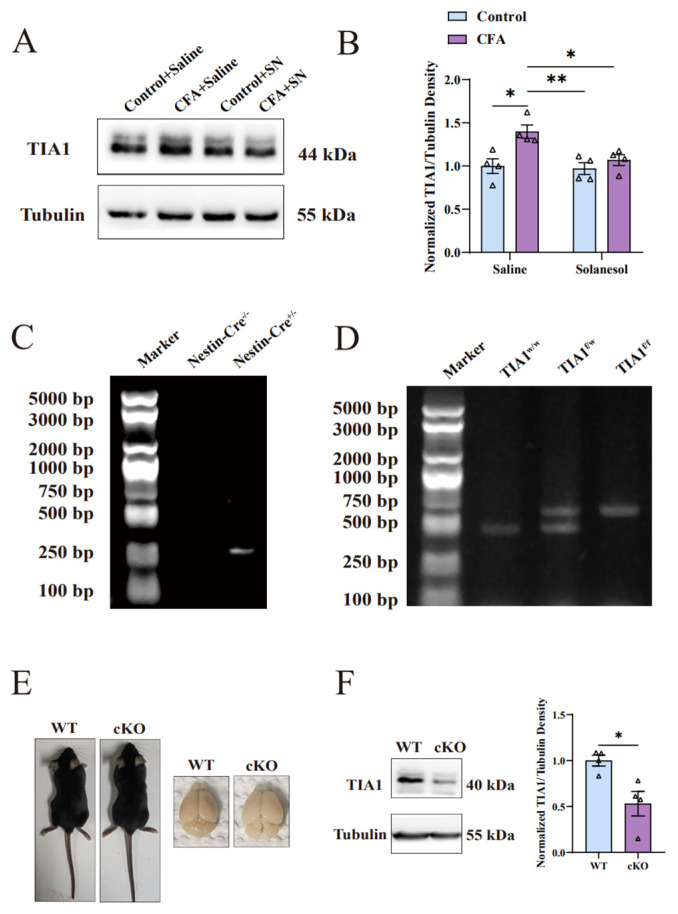
Solanesol decreased the upregulation of TIA1 induced by CFA in the ACC. (**A**,**B**) Representative Western blot analysis results for TIA1 (**A**) and density ratio of TIA1 to β-tubulin (**B**) indicated that solanesol significantly decreased TIA1 expression, which was elevated after CFA injection to approximately the normal level; however, it did not influence the normal TIA1 expression in the control group. Statistical analysis was performed using two-way ANOVA (followed by Bonferroni’s multiple comparisons test). Values are expressed as mean ± S.E.M. (n = 4/group; * *p* < 0.05, ** *p* < 0.01). (**C**,**D**) Comparison of mice with and without the Nestin-Cre gene after polymerase chain reaction (**C**). Comparison of mice with and without TIA1 floxed after polymerase chain reaction (**D**). TIA1^f/f^ represents the homozygote of TIA1-floxed mice. TIA1^f/w^ represents the heterozygote of TIA1-floxed mice. TIA1^w/w^ represents TIA1 wild-type mice. (**E**) Body and brain size comparisons between cKO and wild-type mice. (**F**) Representative Western blot analysis results for TIA1 in the ACC showed that its expression decreased after the conditional knockout compared with the wild type. Statistical analysis was performed using an unpaired *t*-test. Values are expressed as mean ± S.E.M. (n = 4/group; * *p* < 0.05). Abbreviations: ACC, anterior cingulate cortex; ANOVA, analysis of variance; CFA, complete Freund’s adjuvant; TIA1, T cell-restricted intracellular antigen-1; SN, solanesol; cKO, TIA1^Nestin^ knockout; WT, wild-type; kDa, kilodalton; bp, base pair.

TIA1, as previously mentioned, has an inseparable relationship with pro-inflammatory cytokines (i.e., IL-1β and TNF-α). Therefore, we measured the TIA1 expression levels. As expected, we found that the TIA1 level in the ACC significantly decreased in the CFA+solanesol group, whereas it significantly increased in the CFA group (Figure 4A,B). This suggests that TIA1 may be involved in SN’s regulation of anxiety. 

### 2.5. TIA1 cKO Mice Did Not Exhibit Anxiety-like Behaviors after CFA Injection

**Figure 5:**
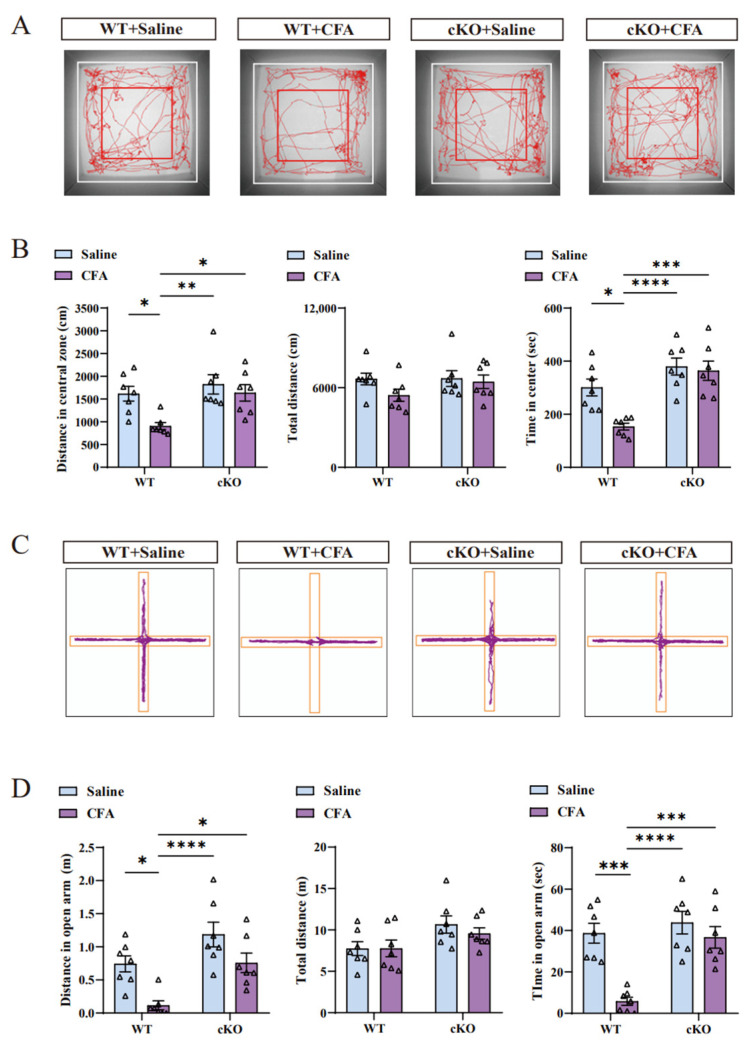
Conditional knockout of TIA1 improved the anxiety-like behaviors induced by CFA injection. (**A**,**B**) Based on the tracking map of the open field test (**A**), cKO mice did not show decreased levels of exploration in the central area after CFA injection. The time and distance in the central zone (**B**) clearly indicate that cKO mice after CFA treatment did not exhibit anxiety-like behaviors compared with control mice after CFA treatment. Statistical analysis was performed using two-way ANOVA (followed by Bonferroni’s multiple comparisons test). Values are expressed as mean ± S.E.M. (n = 7/group; * *p* < 0.05, ** *p* < 0.01, *** *p* < 0.001, and **** *p* < 0.0001). (**C**,**D**) Tracking plot of the elevated plus-maze (**C**) shows that cKO mice did not have decreased motivation with respect to discovering the open arm after CFA injection. The time and distance in the open arm (**D**) prove that cKO mice were not anxious after CFA injection. Statistical analysis was performed using two-way ANOVA (followed by Bonferroni’s multiple comparisons test). Values are expressed as mean ± S.E.M. (n = 7/group; * *p* < 0.05, *** *p* < 0.001, and **** *p* < 0.0001). Abbreviations: ANOVA, analysis of variance; CFA, complete Freund’s adjuvant; cKO, TIA1^Nestin^ knockout; TIA1^Nestin^, TIA1 knockout in Nestin-positive cells; WT, wild-type.

To further confirm the role of TIA1 in alleviating CFA-induced anxiety-like behaviors with solanesol treatment, cKO mice were employed (Figure 4C–F), and the experiment was performed as shown in Figure 1B. In the open field test, the CFA+cKO group spent more time and traveled longer distances in the central zone compared to the WT+CFA group. In the elevated plus-maze test, CFA+cKO mice were more willing to explore the open arm than mice in the WT+CFA group. Similar to the WT+saline group, the CFA+cKO group did not exhibit anxiety-like behaviors after CFA injection (Figure 5A–D). The cKO mice had similar behaviors in the open field and elevated plus-maze test compared with WT mice.

### 2.6. Decreased Activation of Microglia and Astrocytes Was Observed in the ACC of cKO Mice after CFA Injection

**Figure 6:**
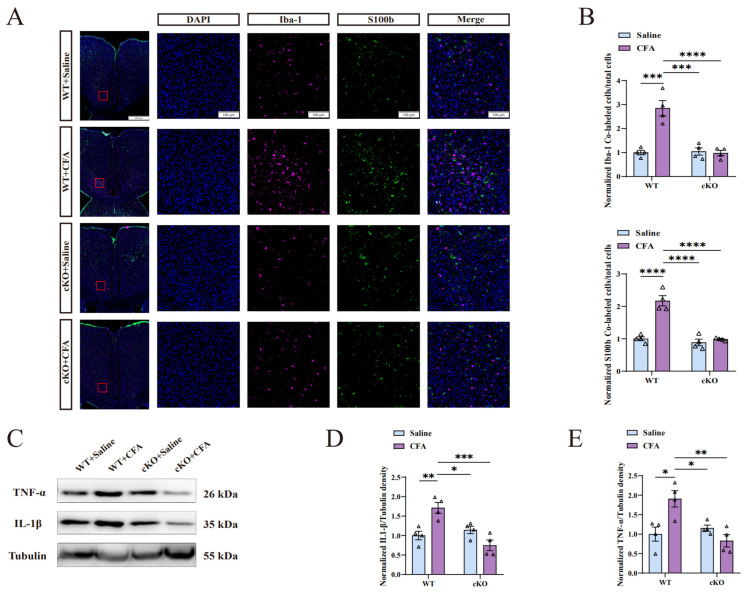
Neuro-inflammation in the ACC of cKO mice did not occur after CFA injection. (**A**) Markers (Iba-1, S100b, and DAPI) used in the second experiment were the same as in the first experiment, as well as the scale bar and significance of the red boxes. (**B**) Microglia and astrocytes in the ACCs of cKO mice that show increased activation after CFA injection were restricted, indicating that the TIA1 knockout in neural stem cells maintained microglial and astrocytic activation at a normal level, similar to that in cKO mice and WT mice without CFA injections, and this confirmed that solanesol plays a role through TIA1 regulation. Statistical analysis was performed using two-way ANOVA (followed by Bonferroni’s multiple comparisons test). Values are expressed as mean ± S.E.M. (n = 4/group; *** *p* < 0.001, **** *p* < 0.0001). (**C**) Representative Western blot analysis results for IL-1β and TNF-α. (**D**) The normalised density ratio of IL-1β to tubulin shows that the knockout of TIA1 resulted in decreased IL-1β expression after CFA injection. Statistical analysis was performed using two-way ANOVA (followed by Bonferroni’s multiple comparisons test). Values are expressed as mean ± S.E.M. (n = 4/group; * *p* < 0.05, ** *p* < 0.01, *** *p* < 0.001). (**E**) Normalized density ratio of TNF-α to β-tubulin shows that the TNF-α levels of cKO mice were maintained at a normal level after CFA injection. Combined with IL-1β density results (**D**), these findings suggest that solanesol ameliorates increased IL-1β and TNF-α levels via TIA1 regulation. Statistical analysis was performed using two-way ANOVA (followed by Bonferroni’s multiple comparisons test). Values are expressed as mean ± S.E.M. (n = 4/group; * *p* < 0.05 and ** *p* < 0.01). Abbreviations: ACC, anterior cingulate cortex; ANOVA, analysis of variance; CFA, complete Freund’s adjuvant; cKO, TIA1^Nestin^ knockout; DAPI, 4′,6-diamidino-2-phenylindole; Iba-1, ionized calcium-binding adapter molecule 1; S100b, S100 calcium binding protein B; TIA1, T cell-restricted intracellular antigen-1; TIA1^Nestin^, TIA1 knockout in Nestin-positive cells; IL, interleukin; TNF-α, tumor necrosis factor α; WT, wild-type; kDa, kilodalton.

TIA1^Nestin^ cKO mice helped to confirm whether TIA1 expression influences microglial and astrocytic activation after CFA injection. Upon the completion of the two behavioral tests, microglial and astrocytic activations in the ACC of CFA+cKO mice were detected via immunofluorescence staining. The results showed that CFA+cKO mice had decreased microglial and astrocytic activation in the ACC after CFA injection, and the ratio of Iba-1-positive and S100b-positive cells was similar to that in the cKO+saline and WT+saline group (Figure 6A,B). 

### 2.7. cKO Mice Exhibited a Decreased Neuro-Inflammation Response in the ACC after CFA Injection

On day 7, after obtaining ACC samples from the cKO+CFA group and the other three groups, we performed Western blot analysis to detect IL-1β and TNF-α levels, which reflect the degree of neuro-inflammation response in the ACC. The IL-1β and TNF-α levels in cKO+CFA mice were similar to those of mice in the WT+saline and cKO+saline groups and were considerably lower than those in the WT+CFA group (Figure 6C–E). This finding indicates that decreased TIA1 expression promotes the downregulation of IL-1β and TNF-α levels in the ACC. Importantly, this finding indicates that solanesol’s mechanism of function is via TIA1 regulation, which mitigates the neuro-inflammation response.

## 3. Discussion

**Figure 7:**
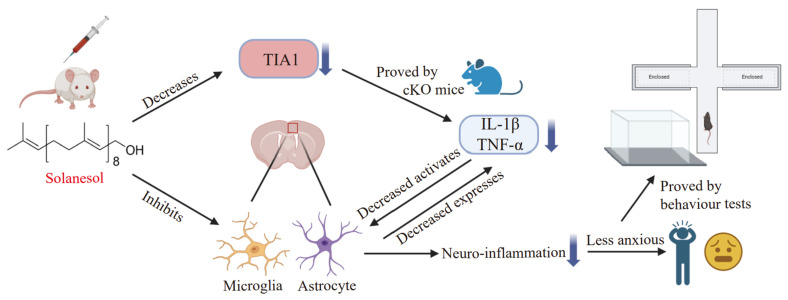
The key mechanisms of solanesol in the alleviation of anxiety. Solanesol can ameliorate anxiety-like behaviors induced by neuroinflammation by inhibiting glial activation and decreasing TIA1 expression, thereby reducing pro-inflammatory cytokines. This figure was created using BioRender.com. Abbreviations: cKO, TIA1^Nestin^ knockout; TIA1, T cell-restricted intracellular antigen-1; IL-1β, interleukin-1β; TNF-α, tumor necrosis factor α.

Anxiety disorder has many potential causes, with neuro-inflammation being a major contributor [25]. In this study, we created a classical mouse model of anxiety-like behaviors induced by neuro-inflammation through hind paw injections of CFA [26]. As expected, CFA injections successfully evoked anxiety-like behaviors, as indicated by the open field and elevated plus-maze test results. Importantly, solanesol treatment ameliorated these anxiety-like behaviors, suggesting its potential as an anxiolytic agent. Our study explored the potential mechanism underlying solanesol’s ability to alleviate anxiety using Western blot and immunofluorescence analyses. We found that solanesol reduced microglial and astrocytic activation in the ACC, a key brain region associated with anxiety and neuro-inflammation. Solanesol’s effects were mediated through the downregulation of TIA1, which in turn decreased the levels of pro-inflammatory cytokines IL-1β and TNF-α. This finding is significant because traditional anxiolytic medications primarily target neuronal pathways, often neglecting the role of microglial activation and inflammation in anxiety (Figure 7). 

Current treatments for anxiety, including selective serotonin (5-HT) re-uptake inhibitors and benzodiazepines, focus on neuronal regulation. However, these drugs do not target the microglia-mediated inflammatory response, which can influence neurons and exacerbate anxiety disorders [7]. Microglial activation reportedly has a deep impact on astrocytes and neurons. The excessive accumulation of IL-1β and TNF-α released by microglial activation stimulates neurons and even causes cell death under chronic neuro-inflammation conditions [8]. In addition, the activation of microglia by releasing cytokines, especially IL-1β, can result in the activation of astrocytes, which exacerbates neuro-inflammation [27]. Our findings indicated that microglial regulation may offer a novel approach to treating anxiety.

The ACC’s role in neuro-inflammation and anxiety disorders is well-documented [28,29]. Thus, we chose to monitor the microglia and astrocytes of the ACC in the immunofluorescence assays and the tissue of the ACC in Western blot analyses in order to identify the potential mechanisms and targets of solanesol. In the Western blot analysis, we first observed that IL-1β and TNF-α levels decreased after solanesol treatment, providing bi-directional evidence of microglial inhibition [16,29,30]. Since TIA1 is an upstream factor of these two cytokines [15], we analyzed its expression. Interestingly, TIA1 expression in the ACC of the solanesol group was significantly lower than that of the CFA group, indicating that solanesol influenced the neuro-inflammation response by regulating TIA1 expression. Thus, we used cKO mice to conduct a further control experiment to determine whether low TIA1 expression levels are associated with solanesol treatment. As expected, cKO mice exhibited low microglial activation and expression levels of the two cytokines after CFA injections, and the anxiety-like behaviors dramatically disappeared. 

Indeed, only a few studies have been published on the role of TIA1 in anxiety-like behaviors. Therefore, the mechanisms of TIA1 in the modulation of anxiety-like behaviors require further study. For example, a previous study confirmed that TIA1 is the translational silencer of TNF-α in the peripheral immune system [31]. In addition, TIA1 and pro-inflammatory cytokine levels are inversely proportional in in vitro experiments [32]. However, the pro-inflammatory cytokine levels in cKO mice after CFA injections were strangely similar to those in normal mice. Indeed, some studies have shown that the relationship between TIA1 and pro-inflammatory cytokines is directly proportional in some conditions [33], indicating that the effect of TIA1 on pro-inflammatory cytokines in the ACC has not been completely clarified. However, we confirmed that these two cytokine levels (IL-1β and TNF-α) decreased as TIA1 levels decreased. The specific mechanism related to the relationship between TIA1 and IL-1β and TNF-α requires further exploration in the ACC. Moreover, the microglia and astrocytes in the cKO+CFA group were not as active compared with those in the WT+CFA group. This finding indicates that the *TIA1* knockout in neural stem cells inhibited microglial and astrocytic activation; this suggests that TIA1 in neural stem cells and cells differentiated from neural stem cells could contribute to IL-1β and TNF-α expression, thereby resulting in the microglia-mediated neuro-inflammation response.

Some researchers have made great contributions, especially from the neuroplasticity-changing aspect [34], in proving that anxiety-like behaviors can arise due to the activation of microglia, which can release pro-inflammatory factors in the ACC. However, the relationship between TIA1 in ACC and anxiety has not been clarified. Notwithstanding quantities of efforts in finding plant-active ingredients such as scopoletin and sophoridine [34,35] for anti-anxiety, some of them are still omitted, such as solansol. Based on the Western blotting and immunofluorescence results, solanesol produces a marked effect through TIA1 regulation, revealing the value of solanesol in treating anxiety, and indicating that TIA1 may be a potential new target for anxiety treatment. 

Considering all the results together, we drew the following conclusions. (1) CFA injections cause a significant neuro-inflammation response by increasing IL-1β and TNF-α levels in the ACC, resulting in severe anxiety-like behaviors. (2) Solanesol treatment ameliorates anxiety-like behaviors by inhibiting the neuro-inflammation response. (3) Solanesol decreases IL-1β and TNF-α levels via TIA1 regulation (specifically by decreasing TIA1). (4) Solanesol inhibits microglial and astrocytic activation. In summary, our study showed that solanesol can improve anxiety-like behaviors induced by CFA injections in mice by modulating TIA1 in the ACC. Further research is needed to evaluate solanesol’s anxiolytic effects in other anxiety models and its potential clinical applications. Drugs targeting TIA1 may offer new avenues for anxiety disorder treatment.

## 4. Materials and Methods

### 4.1. Animals 

In this study, we used male C57BL/6 mice and mice with *TIA1* conditional knockout in Nestin-positive cells (TIA1^Nestin^ cKO) aged 6–8 weeks to conduct experiments. TIA1^Nestin^ cKO mice were generated by crossing heterozygotes of *TIA1* allele floxed (TIA1^f/f^) mice with Nestin-Cre transgenic mice (003771, Jackson Laboratory, Bar Harbor, ME, USA), in which Cre recombinase is expressed in neural stem cells under the control of the Nestin promoter. This creates a conditional knockout of *TIA1* in neural stem cells, as well as their derivatives, including neurons and astrocytes. TIA1^f/f^ mice were obtained by crossing with the heterozygotes of *TIA1* floxed (TIA1^f/w^) mice, which were generated by Shanghai Biomodel Organism Science and Technology Development Co., Ltd. (Shanghai, China). A targeting vector containing the first two exons of the *TIA1* gene was created by recombineering. Briefly, transformed embryonic stem cell (ES) colonies were screened using long-template polymerase chain reaction (PCR) with the following primer sets: P5F (5′-AACCCTTTTTCCACTTGCTTACT-3′) and P5R (5′-GCCTTGCCCCTCATCCA-3′) generated a 6.3 kb band in positive clones and P3F (5′-TTCAGCCAGCAACGAGTCA-3′) and P3R (5′-TGGGGAAAAGGAGGTAACATAGG-3′) generated a 5.5 kb band in positive clones. Successfully targeted ES clones (confirmed by both 5′PCR and 3′PCR fragments) were micro-injected into C57BL/6J blastocysts. Germline transmission from generated chimeric offspring was confirmed using long-template PCR. Mice carrying the targeted allele were bred with flippase recombinase transgenic mice to remove the flippase recognition target-flanked neomycin resistance cassette and generate TIA1^flox^ mice. Genomic DNA extracted from tail biopsies were genotyped with a PCR primer set (P1: 5′-GAGGCATCAGAATTGTTTTAGTG-3′ and P2: 5′-GAGATTCTGCGGGGCGATAG-3′), which generated a 588 bp band from the wild-type (WT) allele and a 677 bp band from the TIA1 floxed allele. Flippase was isolated by crossing TIA1^f/w^ flippase mice with WT mice. Genomic DNA extracted from tail biopsies was genotyped with a PCR primer set (PA: 5′-CACTGATATTGTAAGTAGTTTGC-3′ and PB: 5′-CTAGTGCGAAGTAGTGATCA-GG-3′), which generated no band from the WT allele and a 725 bp band from the flippase allele. All WT, TIA1^f/w^, TIA1^f/f^, Nestin-Cre^+/−^, and cKO mice were maintained in the C57BL/6J strain background. For all experiments, mice (weight, 20–35 g) were housed with four or five mice per cage at a constant room temperature (25 ± 1 °C) and relative humidity (60 ± 5%) under a 12-h light/dark cycle (lights on from 07:00–19:00); food and water were available ad libitum. The mice were adapted to the laboratory conditions for approximately 1 week and habituated to the testing environment for at least 15 min before all experiments and behavioral tests. At least four pairs of mice from the same litter were used in all experiments. Significant effort was carried out to minimize the total number of animals used while maintaining statistically valid group numbers. All experiments involving animals were approved by the Laboratory Animals Ethics Committee of Hangzhou Normal University (approval number: HSD20220104).

### 4.2. CFA Injection and Solanesol Treatment

Mice were administered an intra-plantar injection of CFA (40 μL) into the plantar surface of the left hind paw to induce anxiety-like behaviors. For the solanesol treatment, solanesol was diluted to 5 mg/mL (final concentration, 50 mg/kg [36]) with normal saline via ultrasonication. Solanesol was administered via intra-peritoneal injection at the same time daily. Two experiments were conducted. The first experiment consisted of four groups: control+saline (control group), CFA+saline (CFA group), control+solanesol, and CFA+solanesol. The control+solanesol group was used to confirm that solanesol has no influence on normal mice. The second experiment consisted of four groups: WT+saline, WT+CFA, cKO+saline, and cKO+CFA. In the first experiment, the control+saline group mice received 10 μL/g saline intra-peritoneally and also received an intra-plantar injection of saline (40 μL each). In the second experiment, the WT+saline and cKO+saline groups received an intra-plantar injection of saline (40 μL each). Furthermore, another batch of new WT mice was used for the WT and WT+CFA groups in the second experiment. The final solanesol and saline injections were administered 1 h before the behavior tests on day 7. Figure 1 illustrates the detailed experimental process.

### 4.3. Open Field Test

The open field test was conducted in a 50 × 50 × 50 cm open field chamber with a translucent plexiglass wall and detachable opaque top in which the mice were allowed to explore freely for 30 min. The central area occupied 35% of the total area. Data including time, entries, and distance in the central zone and the total distance traveled during the test were recorded using a digital video camera, and they were analyzed using EthoVision XT 12 (Noldus Information Technology, Beijing, China). The entire apparatus was cleaned at least once using 75% ethanol between experiments.

### 4.4. Elevated Plus-Maze Test

The apparatus for the elevated plus test consisted of two open and two closed arms (5 × 30 cm [each arm], 5 × 5 cm center areas, and a 15 cm wall height for closed arms) and was placed 50 cm off the floor. At the start of the experiment, the head of each mouse was placed toward the center area of one open arm, and the mouse was allowed to explore the maze freely for 5 min, which was recorded via a digital video camera (C270, Logitech, Glasgow, UK). Movements and time spent in different areas were recorded and analyzed using ANY-maze software (Version 7.00, Stoelting Co., Wood Dale, IL, USA). The apparatus was cleaned using 75% ethanol after each experiment.

### 4.5. Western Blot Analysis

Western blot analysis was performed as previously described [21,22]. After completing the behavioral tests, the mice were immediately transferred to the animal operating room. The mice were euthanized by cervical dislocation after isoflurane anesthesia. The ACC regions were dissected and homogenized in an ice-cold radioimmunoprecipitation assay lysis buffer containing phosphatase and protease inhibitors. The total protein content of the collected samples was diluted to a minimum concentration according to the bicinchoninic acid assay using a radioimmunoprecipitation assay lysis buffer. A sodium dodecyl-sulfate loading buffer (5×) was added and diluted to 1× according to the volume of each sample after homogenization. Equal amounts of protein (30 μg) were separated according to molecular weight using sodium dodecyl-sulfate poly-acrylamide gel electrophoresis; then, they were electro-transferred onto poly-vinylidene difluoride membranes. After incubation in 5% skim milk for 1 h at room temperature (26 °C) and washing in 1× Tris-buffered saline with Tween (TBST) (three times, 15 min each), the poly-vinylidene difluoride membranes were incubated with primary antibodies at 4 °C overnight. The following primary antibodies were used: mouse monoclonal anti-β-tubulin (1:1000, cat. 200608, Chengdu Zen Bioscience Co., Ltd., Chengdu, China), rabbit polyclonal anti-TIA1 (1:1000, cat. 12133-2-AP, Proteintech, Rosemont, IL, USA), rabbit polyclonal anti-TNF-α (1:400, cat. GB11188, Wuhan Servicebio Technology Co., Ltd., Wuhan, China), and rabbit polyclonal IL-1β (1:500, cat. GB11113; Wuhan Servicebio Technology Co., Ltd.). After washing in 1× TBST (three times, 15 min each), the membranes were incubated in horseradish peroxidase-conjugated secondary antibodies (1:10,000, cat. A0545 [goat anti-rabbit], A9044 [goat anti-mouse], Sigma Aldrich, St. Louis, MO, USA). Subsequently, the membranes were washed in TBST three times, and protein bands were visualized with an enhanced chemiluminescence detection kit (#FD8020, FDbio, Hangzhou, China). The images were quantified using ImageJ software (Version 2.9.0, National Institutes of Health, Bethesda, MD, USA). To analyze the data, the band intensity of each protein was normalized to the band intensity of β-tubulin. The intensity of the control group was set at 100% whereas the intensities of the other treatment groups were expressed as percentages relative to the control group.

### 4.6. Immunofluorescence

Mice were anesthetized with isoflurane and then transcardially perfused with 0.1 M phosphate-buffered saline (PBS) followed by 4% paraformaldehyde in PBS. Brains were post-fixed in 4% paraformaldehyde overnight at 4 °C. Subsequently, the brains were incubated in 15% sucrose in PBS overnight, followed by incubation in a 30% sucrose solution. ACC sections (20-μm thick) were acquired with a cryostat (NX50, Thermo Fisher Scientific, Waltham, MA, USA). After fixation in 4% paraformaldehyde for 20 min followed by washing in PBS, the sections were blocked and permeabilized with 0.3% Triton X-100 in PBS containing 5% bovine serum albumin at room temperature. The sections were washed in PBS and incubated in anti-bodies against ionized calcium-binding adapter molecule 1 (Iba-1) (1:500, OB-PGP049-01, Oasis, Hangzhou, China) and S100 calcium-binding protein B (S100b) (1:500, OB-PRB050-01, Oasis) overnight at 4 °C in 5% bovine serum albumin. The sections were then washed and incubated with Alexa647-conjugated goat anti-rabbit (1:1000, A0468, Beyotime, Shanghai, China) and Alexa488-conjugated goat anti-guinea pig (1:1000, OB-GP488-50, Oasis) secondary antibodies and 4′,6-diamidino-2-phenylindole (DAPI) (1:1000) for 1 h. The primary antibodies, secondary antibodies, and DAPI were all diluted in a solution containing 5% bovine serum albumin. The sections were mounted after washing in PBS and photographed under a fluorescence microscope (VS200, Olympus, Tokyo, Japan).

### 4.7. Statistical Analysis

Data were analyzed and plotted using GraphPad Prism 8.0 (GraphPad Software, San Diego, CA, USA). Statistical comparisons were performed using Student’s *t*-test, one-way analysis of variance (ANOVA), two-way ANOVA or two-way repeated measures ANOVA (Tukey’s, Bonferroni’s, or Sidak’s tests were used for post hoc comparisons), or the Kruskal–Wallis test (with Dunn’s post hoc multiple comparisons test). Data are presented as mean ± S.E.M. Statistical significance was set at *p* < 0.05.

## Data Availability

All relevant data are available and can be obtained upon request from the corresponding authors.
